# Medication adherence in adolescents: physiological, psychosocial, and clinical perspectives

**DOI:** 10.1007/s00210-026-05122-1

**Published:** 2026-03-02

**Authors:** N. Ipek Kirmizi Sonmez, Narin Akici, Ahmet Akici

**Affiliations:** 1 Department of Pharmacology, School of Pharmacy, Bahcesehir University, Istanbul, Turkey; 2 Department of Pediatrics, Haydarpasa Numune Training and Research Hospital, Istanbul, Turkey; 3 Department of Medical Pharmacology, School of Medicine, Marmara University, 34854 Maltepe, Istanbul, Turkey

**Keywords:** Adolescents, Medication, Adherence, Child health, Rational pharmacotherapy

## Abstract

Adolescence is a developmental stage characterized by significant physiological, psychological, and social changes. These changes are known to have a direct impact on medication use and adherence to treatment. Adolescents tend to have low adherence rates to treatment for chronic illnesses. When irrationally managed, this situation can lead to problems such as inadequate disease control, increased risk of complications, and increased use of healthcare services. The aim of this review is to evaluate the factors affecting medication adherence in adolescents, especially those specific to adolescents, considering the current literature and to suggest solutions. This review examines the implications of physiological and neurodevelopmental differences accompanying pubertal development on pharmacokinetic and pharmacodynamic processes. It also discusses the role of psychosocial factors such as the pursuit of autonomy, peer influence, aesthetic concerns, and performance anxieties on adherence during adolescence. Furthermore, changes in drug formulations and routes of administration, increased independence in accessing medications, and the transfer of treatment responsibility from parents to adolescents are evaluated. The potential contributions of digital health applications and mobile technologies to supporting adherence in adolescents are also addressed. Finally, recommendations aimed at increasing adherence through rational drug use in adolescents are presented. In conclusion, drug adherence in adolescents, which significantly determines the outcome of pharmacotherapy, is a complex process shaped by the interaction of numerous biological and psychosocial factors that requires holistic, developmentally appropriate approaches.

## Introduction

Childhood is a period comprising distinct developmental stages, extending from birth to the completion of puberty and encompassing individuals under 18 years of age. The phase that begins at approximately 10–12 years of age and continues until the end of childhood is defined as adolescence (World Health Organization 2001; Sawyer et al. 2018; Balasundaram and Avulakunta 2025). From the perspective of medication use, childhood represents a highly distinctive period characterized by physiological, pharmacokinetic, pharmacodynamic, and psychosocial features that differ from those of adults, while also encompassing age-related variability within itself. These differences have provided a basis for pediatric medication use to be frequently addressed and discussed in the scientific literature (World Health Organization 2007; Smits et al. 2022; Watanabe et al. 2024). Moreover, some of these potential challenges become even more pronounced in the adolescent population (Taddeo et al. 2008).

According to the World Health Organization (WHO), adherence is defined as the extent to which an individual’s behavior such as medication use, adherence to dietary recommendations, and lifestyle modifications corresponds with the agreed recommendations of a healthcare professional (World Health Organization 2003). Medication adherence in adolescents, particularly in the context of chronic diseases, is lower than in other stages of childhood. The primary reasons for this include adolescent-specific autonomy seeking, questioning of treatment necessity, peer influence, the perceived restrictions imposed by treatment on daily life, and various psychological and environmental factors (Kyngas et al. 2000; World Health Organization 2007; Taddeo et al. 2008; Desai and Oppenheimer 2011; Hanghøj and Boisen 2014). Reduced adherence in this age group increases the risk of disease-related complications, complicates disease management and follow-up, decreases the effectiveness of healthcare services, and leads to higher treatment costs (Farre et al. 2025). Therefore, improving medication adherence among adolescents should be considered a priority area requiring careful attention within pediatric healthcare services (Taddeo et al. 2008; Hanghøj and Boisen 2014).

While pediatric pharmacotherapy has been widely studied, the literature addressing its limitations has predominantly focused on younger age groups or the general pediatric population, rather than adolescents as a distinct developmental group (World Health Organization 2007; Kimland and Odlind 2012; European Commission 2017; Akıcı et al. 2020; Samuels et al. 2023). Treatment adherence among adolescents with chronic diseases varies according to the type of condition and the medications used; however, it has consistently been reported to be markedly lower than in childhood, with non-adherence rates reaching up to 80% in certain cases. Importantly, adherence estimates in this age group depend strongly on the assessment method used—such as self-report, caregiver report, pharmacy refill data, electronic monitoring, or biological markers—each with distinct strengths and limitations. Therefore, high non-adherence rates should be interpreted considering the measurement methodology rather than as directly comparable absolute figures (Rapof 2010).

The aim of this narrative review is to evaluate medication adherence in adolescents considering the existing evidence, to highlight challenges that are specific to adolescence and distinct from those observed in earlier childhood, and to discuss practical strategies to support rational pharmacotherapy and improve adherence in this population. In this regard, the literature was identified through targeted searches of PubMed, Web of Science, and Google Scholar, focusing primarily on publications from 2000 to 2025, using combinations of keywords such as “adolescent, medication adherence, non-adherence, pharmacokinetics, pharmacodynamics, psychosocial factors, and transition to adult care.” Priority was given to high-quality reviews, clinical guidelines, consensus statements, and seminal original studies relevant to adolescent pharmacotherapy and adherence.

### A conceptual framework for determinants of medication adherence in adolescents

To organize the diverse factors influencing medication adherence during adolescence, we adopt a conceptual framework that categorizes determinants across five interacting levels: developmental and physiological factors, therapy-related factors, healthcare system and care process related factors, social context and society related factors, and disease related factors. This framework is used throughout the review to structure the discussion and to highlight adolescent-specific modifiers at each level.

## Developmental and physiological factors

Adolescence is a critical transitional period characterized by rapid growth and development, serving as a bridge between childhood and adulthood (Rosen 2004). The developmental and physiological changes that occur during this stage, as detailed below, may influence drug responses from both pharmacokinetic and pharmacodynamic perspectives (Kearns et al. 2003; Smits et al. 2022; Watanabe et al. 2024).

- **Changes in growth and body composition**: During adolescence, linear growth and increases in muscle mass accelerate, while sex-specific differences in body fat distribution become more pronounced. Fat mass proportion increases in females, whereas males exhibit marked increases in muscle mass and lean body mass. These changes may influence drug pharmacokinetics in an age- and sex-dependent manner, particularly by accentuating the differentiating role of lipophilic versus hydrophilic drug properties, and by affecting the volume of distribution and other pharmacokinetic parameters (Kearns et al. 2003; Wood et al. 2019; Smits et al. 2022). Consequently, dose and dosing interval adjustments based on pharmacokinetic characteristics may be required in adolescents with markedly increased muscle or fat mass. In addition, physiological increases in serum creatinine levels may be observed, particularly in males and adolescents with higher muscle mass (Baxmann et al. 2008). This consideration should be considered in the management of conditions that require creatinine monitoring.
- **Endocrine changes:** During adolescence, pronounced changes occur in the hypothalamic–pituitary–gonadal axis. With the onset of puberty, sex-specific increases are observed in gonadotropins, estrogen, progesterone, and testosterone levels. As a result of fluctuations in these hormones and changes originating from the adrenal axis, body composition begins to assume its new configuration and secondary sexual characteristics develop, including the onset of the menstrual cycle in females (Wood et al. 2019). Increases in estrogen and testosterone levels may lead to alterations in the metabolism of certain drugs. The variability of these hormonal changes according to the adolescent’s age and sex further complicates the management of pharmacotherapy (Wood et al. 2019). Rising levels of growth hormone and insulin-like growth factor-1 (IGF-1) alter metabolism and energy utilization (Rosen 2004; Alotaibi 2019). In addition, changes in thyroid hormones, melatonin, serotonin, and dopamine levels may be associated with an increased frequency of conditions such as autoimmune thyroiditis, nutritional disturbances, sleep dysregulation, and mood changes (Soldin and Mattison 2009; Blakemore et al. 2010). These hormonal alterations may also influence the pharmacokinetic properties of medications used both for hormone-related conditions and for other therapeutic purposes (Soldin and Mattison 2009; Smits et al. 2022).
- **Urogenital changes:** The alterations observed in estrogen, progesterone, and testosterone levels observed during adolescence, as discussed under endocrine changes, largely exert their effects on the urogenital system (Wood et al. 2019). Variability in adaptation to these hormonally driven changes and the recognition and management of related disturbances require particular attention. Besides, adolescence may coincide with the onset of sexual activity; it is essential that adolescents are adequately informed about sexually transmitted infections, their prevention and treatment, pregnancy, contraception, pregnancy termination, and teratogenicity (Ott et al. 2025). For example, adolescents should be counseled regarding the potential adverse effects and drug–drug interactions of oral contraceptives, which may be commonly used during this period for various indications. These agents may interact with several drug classes, including antiepileptics, potentially altering their efficacy or adverse effect profiles (Reddy 2010; Jensen and Lara-Torre 2024). Isotretinoin, which is frequently used for acne treatment during adolescence, has well-established teratogenic properties and other critical adverse effects that should be clearly and thoroughly explained to patients. In addition, changes in vaginal and vulvar microbiota and a reduction in vaginal pH may predispose adolescents to new infections or lead to altered effectiveness of certain agents, such as azole antifungals, due to increased acidity (Danby et al. 2012; Hickey et al. 2015). Furthermore, estrogen-mediated thinning of cervical mucus may increase the absorption of some intravaginally administered medications (Holt et al. 1979).
- **Changes in hepatic and renal function:** During adolescence, alterations in hepatic enzyme activity and bile salt concentrations are observed. In particular, non-linear changes in cytochrome P450 enzyme activity may lead to clinically significant differences in the metabolism of certain drugs compared with childhood and adulthood (Kennedy 2008). In parallel, increases in renal blood flow, glomerular filtration rate, and tubular secretion may affect drug elimination processes (Fernandez et al. 2011; Watanabe et al. 2024).
- **Neurological changes: **The delayed maturation of the prefrontal cortex, ongoing development of cognitive capacity, earlier activation of the limbic system, and increased dopaminergic activity together constitute a neurobiological basis for heightened emotional variability and risk-taking behavior during adolescence. These changes may influence not only behavioral characteristics but also motivation toward medication use and adherence (Blakemore et al. 2010; Sawyer et al. 2018; Farre et al. 2025). This process may manifest as denial of illness, postponement of medication intake, underestimation or irregular use of prophylactic treatments, and premature discontinuation of therapy (Farre et al. 2025). In this neurodevelopmental context, increased dopaminergic activity within the limbic system may further shape reward processing and motivational priorities, leading adolescents to prioritize immediate rewards over long-term health benefits. Consequently, factors such as short-term relief or social acceptance may undermine motivation to adhere to treatment regimens.
  Moreover, physiological changes in circadian rhythm and sleep patterns during adolescence may indirectly affect adherence by leading to missed or irregular medication dosing times (Hanghøj and Boisen 2014). For instance, antiepileptic drugs that are prescribed for morning administration may not be taken consistently due to late bedtimes and delayed awakening, while medications intended for use at bedtime may be taken irregularly due to sleep disturbances (Hofstra et al. 2012).
- **Psychiatric and psychosocial changes:** During adolescence, processes such as identity formation, the pursuit of autonomy, and the increasing prominence of social relationships constitute psychosocial dynamics characterized by intense emotional fluctuations that directly shape health-related behaviors (Campagna et al. 2020; Farre et al. 2025). The literature indicates that approximately one quarter of adolescents experience at least one mental disorder (Olfson et al. 2015; Campagna et al. 2020). The most commonly reported conditions include attention-deficit/hyperactivity disorder (ADHD), anxiety disorders, mood disorders, and behavioral disorders (Kyngas et al. 2000). Mental health disorders have also been shown to substantially impair treatment adherence. In particular, comorbid conditions such as depression, anxiety, and ADHD have been associated with a higher likelihood of reduced medication adherence (Taddeo et al. 2008; Hanghøj and Boisen 2014; Campagna et al. 2020; Elhosary et al. 2023). For example, adolescents with depression may perceive treatment as “meaningless,” whereas individuals with ADHD frequently experience difficulties in maintaining regular medication use (Edgcomb and Zima 2018).

In addition, adolescence is commonly characterized by an increase in psychiatric symptoms, risk-taking behaviors, boundary-testing tendencies, and resistance to parental authority. These behaviors may contribute to higher rates of cigarette smoking, alcohol consumption, and substance use. The use of such substances may negatively affect adherence through mechanisms such as impaired attention, forgetfulness, and decreased motivation. Moreover, the potential of these substances to alter the pharmacokinetic and pharmacodynamic properties of medications may directly influence the efficacy and safety of drugs used by adolescents (National Institute of Drug Abuse 2011; Azar et al. 2024).

Adolescence represents a critical developmental stage during which experimentation with tobacco, alcohol, and other substances often begins, alongside heightened identity exploration, peer influence, academic pressures, and oppositional behavior toward authority figures (Taddeo et al. 2008). These factors may increase the risk of psychiatric conditions such as behavioral disorders, anxiety, depression, and substance use disorders, frequently necessitating pharmacological treatment (Olfson et al. 2015). However, adolescence is also a period in which challenges related to medication adherence, dosing errors, and safe treatment management may become more pronounced. Within this context, suicidal behavior represents an important but relatively infrequent clinical risk that requires careful and proportionate attention. Some medications accessible to adolescents may be involved in suicide attempts, and an increased risk of suicidal ideation and behaviors has been reported particularly during the early phases of antidepressant treatment. Current clinical guidance therefore emphasizes routine risk assessment, close monitoring during the initial months of treatment, education of families and caregivers regarding warning signs, and, when appropriate, measures such as safe storage of medications and limiting dispensed quantities. Combining pharmacotherapy with psychotherapy is essential, particularly for adolescents with depressive disorders, to support both treatment effectiveness and safety. In addition, addressing stigma surrounding suicidal thoughts and behaviors in a sensitive and non-alarmist manner is necessary to promote open communication, treatment continuity, and patient safety (World Health Organization 2014; Food and Drug Administration 2018).

## Therapy-related factors

### Pharmacokinetic and pharmacodynamic changes affecting medication adherence in adolescents

Adolescence is characterized by rapid physiological and biochemical maturation that affects how medications are absorbed, distributed, metabolized, eliminated, and how they exert their effects (Watanabe et al. 2024). While these pharmacokinetic and pharmacodynamic changes do not directly determine whether medications are taken, they can influence adherence indirectly by modifying clinical response, tolerability, and dosing requirements. Consequently, particular attention must be paid to pharmacotherapy during the transition from childhood to adulthood, taking these critical changes into account.

#### Pharmacokinetic changes

- **Absorption:** During adolescence, the gastrointestinal system largely reaches adult functional levels. As gastric emptying time, intestinal motility, and gastric pH become comparable to those of adults, drug absorption is generally more predictable than in earlier childhood (Kearns et al. 2003; Smits et al. 2022). Nevertheless, changes in dietary habits—such as irregular meals, consumption of caffeinated beverages, and the use of tobacco or alcohol—may indirectly affect absorption and bioavailability. For example, the bioavailability of iron preparations may be reduced in adolescents due to the frequent consumption of tea, coffee, carbonated beverages, and fast food (Piskin et al. 2022), potentially contributing to perceived treatment inefficacy. In adolescence, particularly among males, increases in muscle mass may affect the absorption characteristics of medications administered via the intramuscular route. In this route of administration, greater muscle mass and the relatively rich vascularization of muscle tissue may facilitate more rapid entry of the drug into the systemic circulation. In contrast, interindividual variability in muscle mass and increased subcutaneous fat tissue—especially in adolescents with obesity—may result in inadvertent subcutaneous rather than intramuscular injection when inappropriate needle length or technique is used, leading to delayed or variable absorption and reduced pharmacokinetic predictability (Chillistone and Hardman 2017; Poliania Gutierrez and Munakomi 2025). Puberty-associated increases in physical activity and alterations in muscle perfusion may further modify absorption kinetics (Lenz and Ma 2016). While these biological factors primarily affect drug exposure rather than medication-taking behavior itself, variability in onset of action or clinical response may indirectly influence adherence through perceptions of inefficacy or inconsistent benefit. Accordingly, careful consideration of age, muscle development, and body composition is essential when selecting intramuscular administration strategies in adolescents
- **Distribution:** Significant changes in body composition are observed during adolescence (Kearns et al. 2003; Rakhmanina and van den Anker 2006). With pubertal development, increases in fat mass—particularly in females—and increases in muscle mass—particularly in males—may alter the volume of distribution of lipophilic and hydrophilic drugs (Baxmann et al. 2008; Wood et al. 2019). An increase in adipose tissue may lead to greater sequestration of lipophilic drugs within fat stores, thereby expanding their volume of distribution, whereas the distribution of hydrophilic drugs may vary according to changes in total body water and muscle mass (Kearns et al. 2003; Rakhmanina and van den Anker 2006; Smits et al. 2022). Increases in sex hormone levels represent another important factor that indirectly influences volume of distribution. Estrogen and progesterone affect fat distribution and fluid balance, potentially altering the tissue distribution of lipophilic drugs, while testosterone-related increases in muscle mass may influence the distribution characteristics of hydrophilic agents (Soldin and Mattison 2009; Smits et al. 2022). In addition, pubertal hormones may affect plasma protein synthesis, leading to changes in albumin and α1-acid glycoprotein concentrations and binding capacity. Such alterations may modify the free fraction of drugs, thereby influencing tissue distribution and clinical response (Soldin and Mattison 2009). Although such distributional changes primarily affect drug exposure rather than medication-taking behavior itself, variability in efficacy or adverse effect profiles may shape adolescents’ treatment experiences and, in turn, indirectly influence adherence through perceived lack of benefit or tolerability concerns. For example, oral contraceptives may exhibit a distinct distribution profile during adolescence due to increased adipose tissue and altered protein-binding characteristics, resulting in changes in efficacy and adverse effect profiles (Rakhmanina and van den Anker 2006).
- **Metabolism:** Age- and sex-related differences in the activity of the cytochrome P450 enzyme family are well recognized (Kennedy 2008; Aljohmani and Yildiz 2025). For instance, higher CYP3A4 activity has been reported in females, whereas increased activity of CYP2D6, CYP2E1, and CYP1A2 has been observed in males (Aljohmani and Yildiz 2025). During adolescence, however, pubertal hormones may induce non-linear fluctuations in metabolic enzyme activity, resulting in elimination rates of certain drugs that differ from those observed in childhood or adulthood (Soldin and Mattison 2009; Smits et al. 2022; Watanabe et al. 2024). In particular, the pharmacokinetic profiles of drugs that undergo extensive hepatic metabolism—such as theophylline, carbamazepine, phenytoin, and phenobarbital—may exhibit clinically meaningful pharmacokinetic changes during adolescence (Kennedy 2008). In addition, pharmacogenetic variability related to drug-metabolizing enzymes may become more apparent in this period (Wehry et al. 2018). Collectively, these factors may contribute to increased rates of perceived inefficacy or adverse effects, which can further indirectly exacerbate problems related to medication adherence.
- **Excretion:** Renal function approaches adult levels during adolescence (Watanabe et al. 2024). Increases in glomerular filtration rate and tubular secretion may lead to more rapid elimination of certain medications. For example, aminoglycoside antibiotics may be eliminated more rapidly in adolescents, which may necessitate increased dosing frequency. Moreover, sex-related differences in drug elimination—such as higher rates of glomerular filtration and tubular secretion in males compared with females—are expected to become more pronounced during this period (Aljohmani and Yildiz 2025). Although these developmental differences primarily affect drug clearance and systemic exposure rather than medication-taking behavior itself, subtherapeutic drug levels or fluctuating clinical responses may influence adolescents’ perceptions of treatment effectiveness.

#### Pharmacodynamic changes

Pharmacodynamic changes during adolescence may influence drug efficacy and adverse effect profiles and, indirectly, treatment adherence. Puberty-related hormonal fluctuations can alter receptor density and sensitivity, while ongoing maturation of neurotransmitter systems may contribute to interindividual variability in drug response (Blakemore et al. 2010; Wahlstrom et al. 2011; Smits et al. 2022).

Physiological changes in sex and thyroid hormones may further modify pharmacodynamic responses to medications such as antidepressants, antipsychotics, and insulin preparations (Azar et al. 2024; Watanabe et al. 2024). For example, a transient reduction in insulin sensitivity during puberty may lead to perceived treatment inefficacy if dosing is not appropriately adjusted (Azar et al. 2024). Similarly, age-related differences in target tissue sensitivity—such as changes in airway responsiveness—may result in asthma treatment responses that differ from those observed in adults (Tantisira et al. 2008).

Ongoing central nervous system maturation may also influence the tolerability of centrally acting drugs, with adverse effects such as sedation, weight gain, or appetite changes being more prominent in some adolescents (Smits et al. 2022; Watanabe et al. 2024). While these pharmacodynamic differences primarily affect clinical response rather than medication-taking behavior itself, experiences of reduced efficacy or intolerable adverse effects may indirectly influence adherence. An increased risk of suicidal ideation during early treatment with selective serotonin reuptake inhibitors further underscores the need for careful monitoring in this population (Food and Drug Administration 2018).

Collectively, these developmental factors indicate that pharmacotherapy in adolescents—particularly with respect to dose selection—cannot be approached solely using a “small adult” paradigm. Individual variability and developmental stage must be considered, with strategies such as individualized dose titration, therapeutic drug monitoring when indicated, and closer clinical follow-up being particularly important in complex cases (Kang and Kim 2019; Noel et al. 2021).

### Changes in dosage forms and routes of administration in adolescents

One of the most important factors limiting medication use during childhood is the applicability and acceptability of pharmaceutical dosage forms. In early childhood, liquid formulations (such as syrups, suspensions, and drops) are frequently preferred due to swallowing difficulties, taste sensitivity, and limited cooperation (European Medicines Agency 2006). In addition, fear of injections and low tolerance for invasive procedures further restrict the use of parenteral routes. With the transition to adolescence, these limitations partially change. As swallowing reflexes mature and cooperative capacity increases, solid dosage forms such as tablets and capsules become more commonly acceptable (European Medicines Agency 2006). Nevertheless, it should be borne in mind that some adolescents in the transitional phase—particularly those who have not fully overcome swallowing difficulties or who exhibit extreme body muscle or fat mass—may still experience substantial challenges with the use of orally administered solid forms or intramuscular injections. Moreover, in adolescence ease of use and regimen simplicity are key determinants of adherence. Medications requiring frequent dosing are more likely to be forgotten, and complex administration schedules may interfere with daily routines. Therefore, long-acting or sustained-release formulations should be preferred when clinically appropriate, and treatment regimens should be kept as simple and practical as possible. Aligning medication administration times with the adolescent’s daily routine may further support consistent use (Hanghøj and Boisen 2014; Farre et al. 2025).

With respect to routes of administration, medications that are commonly administered orally or, when necessary, rectally during preschool and early childhood are increasingly replaced during adolescence by oral (tablet, capsule), inhaled, transdermal, and, when required, parenteral routes. Reluctance toward rectal administration becomes more pronounced in this period. In chronic conditions such as asthma, diabetes, and epilepsy, more advanced delivery methods—including inhaler devices, insulin injections, and transdermal patches—may be favored due to their suitability for independent use by adolescents (European Medicines Agency 2006). While this independence may enhance adherence, it may also exacerbate adherence problems in individuals with insufficient knowledge or skills (Azar et al. 2024). For example, proper shaking of suspensions prior to use or correct inhaler technique is critical for treatment effectiveness. It is therefore essential that details related to dosage forms and routes of administration are explained to adolescents in an age-appropriate manner by healthcare professionals and caregivers, and that comprehension is ensured. When treatment failure or unexpected adverse effects occur, healthcare professionals should always consider potential adherence-related issues.

## Healthcare system and care process related factors

Access to medications during adolescence follows a different dynamic compared with childhood. In childhood, medication use is generally supervised by parents, caregivers, or guardians and occurs primarily through prescription-based products under the guidance of healthcare professionals. In contrast, with the increasing pursuit of autonomy during adolescence, individuals tend to access both prescription and over-the-counter (OTC) medications more independently (World Health Organization 2007; Poyraz Fındık et al. 2016).

With regard to prescription medications, although adolescents are still legally required to be under parental supervision, responsibilities such as prescription renewal, medication procurement, and maintenance of treatment continuity are often partially transferred to the adolescent, particularly among those with chronic diseases (White et al. 2023; Farre et al. 2025). This transfer may occur more abruptly in adolescents living away from their families or in those who marry or become parents at a young age. While increased adolescent-centered access to and use of medications may enhance active participation in one’s own treatment, it may also create a context conducive to increased adherence problems, as illustrated above (Campagna et al. 2020).

The frequency of self-medication practices increases with age during adolescence. When OTC medications, traditional herbal products, dietary supplements, cosmetics, and other health-related products are used without adequate caution (particularly in settings where regulatory oversight is limited or where access and promotion are easy), adolescents are more likely to select and use these products independently and without control, thereby increasing the risk of adverse outcomes (World Health Organization 2007; Shehnaz et al. 2014; Poyraz Fındık et al. 2016). Analgesics, cold and flu preparations, antihistamines, and vitamin–mineral supplements are among the most commonly used OTC products in this age group (Shehnaz et al. 2014; Lee et al. 2017; Liu et al. 2024). Peer influence and social media appear to play a more prominent role than family members in adolescents’ access to these products (Shehnaz et al. 2014). Despite their non-prescription status, irrational use of these agents is well known to cause adverse effects and drug–drug interactions (Lee et al. 2017). For example, uncontrolled use of analgesic, antipyretic, or anti-influenza preparations—many of which contain paracetamol—has been associated with serious hepatotoxicity (Gulmez et al. 2015). The sedative effects of antihistamines may pose significant risks, particularly in countries where individuals are legally permitted to obtain a driver’s license from the age of 16 (Ten Eicket et al. 2001). In addition, some OTC medications may be subject to misuse by adolescents; for instance, a study conducted in the United States reported that 6.6% of high school seniors used cough syrups containing dextromethorphan for their psychoactive or euphoric effects (National Institute of Drug Abuse 2011).

Many sources define adolescence as extending up to 18–19 years of age (World Health Organization 2001; Farre et al. 2025). However, from the age of 18, individuals are legally considered adults, gaining rights such as legal autonomy, independent living, and expanded social engagement (Poyraz Fındık et al. 2016). This transition may substantially weaken parental and healthcare professional–based control mechanisms related to medication use. Therefore, adolescence should be regarded not only as a period of biological and psychosocial maturation but also as a phase during which access to medications and responsibility for treatment should be gradually supported and transferred (Campagna et al. 2020). During this process, it is critical that healthcare professionals and families progressively convey information and responsibility regarding medication access, appropriate use, storage conditions, and potential risks to adolescents in order to ensure sustained adherence (Campagna et al. 2020; Farre et al. 2025).

Considering the medical, social, and legal dimensions of the issue, it is important to define age thresholds for access to medications within the framework of adolescents’ cognitive development and capacity for discernment. In this sensitive context, two alternative approaches to age-based medication access may serve as guiding frameworks (Poyraz Fındık et al. 2016). In the first proposed approach, a critical age threshold of 16 years may be established, supported by closer and more continuous engagement with healthcare professionals and greater utilization of technological tools. Accordingly, individuals aged 16 years and older may be granted access to medications in a manner similar to adults, preferably under the supervision of adult caregivers. Access to medications for adolescents aged 12–15 years may be evaluated based on their level of acquired behavioral self-regulation, allowing the direction of the approach to be determined on an individual basis. Children under the age of 12, however, would not be permitted independent access to medications. The second approach adopts a more protective framework, proposing a critical age threshold of 18 years. Within this model, individuals aged 16–17 years may access medications based on their capacity for behavioral self-regulation and preferably under adult supervision, whereas those under 16 years would not be allowed independent access. Failure to adopt such structured approaches to age thresholds may expose adolescents to more complex and risky situations in settings where guiding frameworks are lacking. Moreover, to prevent or minimize the potential risks associated with each proposed approach, it is essential to consider national and local contexts and to implement appropriate complementary measures. These measures should extend beyond medication purchase and procurement to also encompass conditions of use and should be addressed within a comprehensive, stakeholder-inclusive framework (Poyraz Fındık et al. 2016; Campagna et al. 2020; Farre et al. 2025).

## Social context and society related factors

The social environment and peer relationships play a central role in shaping medication adherence during adolescence (Sansom-Daly et al. 2012; Farre et al. 2025). Concerns that medication use may be perceived by peers as a marker of being “different” or “ill,” as well as anxiety about reinforcing a “patient identity,” may undermine self-esteem and contribute to treatment avoidance (Azar et al. 2024). In this context, visibility and ease of use often outweigh considerations related to taste or formulation; dosage forms that can be easily integrated into daily life and do not draw attention in peer settings are therefore particularly important for supporting adherence.

Given adolescents’ high level of integration with digital technologies, they represent a key target group for the use of digital health interventions aimed at improving adherence. Mobile applications, smart devices, and wearable technologies can support adherence through functions such as medication reminders, treatment monitoring, and motivational reinforcement (Campagna et al. 2020; Farre et al. 2025). Several condition-specific interventions have demonstrated potential benefits; for example, a game-based application grounded in health behavior theories has been developed to support treatment organization and habit formation in adolescents with chronic gastrointestinal diseases (Mehta et al. 2021), and a mobile application has been designed to improve adherence among adolescents receiving epilepsy treatment (Tall et al. 2022). However, evidence for the effectiveness of app-based adherence interventions in real-world adolescent populations remains mixed. Sustained engagement over time is a common challenge, with declining use frequently observed after initial adoption. Moreover, the effectiveness of digital tools may vary according to socioeconomic context, digital literacy, and access to technology. Concerns related to data privacy, security, and confidentiality are particularly salient in adolescence, and monitoring technologies may be perceived as intrusive or controlling during a developmental period characterized by heightened sensitivity to autonomy and surveillance (Akhtar et al. 2022; Livieri et al. 2025). When appropriately selected and integrated into clinical care pathways, clinician-endorsed digital tools may nonetheless offer meaningful benefits. In chronic conditions requiring continuous monitoring, such as type 1 diabetes, glucose-tracking applications and continuous glucose monitoring (CGM) systems can support adolescents’ gradual assumption of responsibility for disease management and facilitate individualized treatment adjustments through real-time data sharing with families and healthcare professionals (Kebede and Pischke 2019; Ehrmann et al. 2022). In contrast, commercially available applications and social media platforms that lack clinical governance may expose adolescents to misinformation regarding medication use, promote risky health behaviors, or encourage inappropriate self-medication practices. The widespread availability of substance-promoting content and unverified health advice on digital platforms thus represents a potential threat to safe medication use and adherence in this age group (Haß and Seifert 2025).

Within this social context, it is clinically important to distinguish between intentional and unintentional non-adherence, as social dynamics influence these patterns in different ways. Intentional non-adherence often reflects a deliberate choice shaped by concerns about peer stigma, body image, perceived loss of autonomy, or a desire to avoid a “patient identity,” particularly when medications are associated with visible use, weight gain, or other socially sensitive adverse effects. In contrast, unintentional non-adherence is more commonly linked to contextual and cognitive challenges typical of adolescence, such as irregular daily routines, sleep–wake cycle shifts, school-related demands, forgetfulness, and emerging executive function limitations. This distinction has practical implications: intentional non-adherence is more likely to respond to shared decision-making, normalization of socially sensitive side effects, and negotiated autonomy in treatment planning, whereas unintentional non-adherence may be better addressed through regimen simplification, alignment of dosing schedules with daily activities, and the use of reminder systems or supportive digital tools. Recognizing these distinct mechanisms helps avoid uniform adherence interventions and allows for more developmentally appropriate and effective management strategies (Taddeo et al. 2008; Campagna et al. 2020; Farre et al. 2025).

## Disease-related factors

The characteristics of the underlying condition are major determinants of medication adherence during adolescence. Acute and chronic illnesses pose distinct challenges, shaped by differences in symptom dynamics, treatment duration, and perceived necessity of therapy. In adolescents, these disease-related factors interact with developmental features such as evolving risk perception, autonomy seeking, and sensitivity to social context, thereby influencing adherence behavior.

Acute conditions, particularly infectious diseases, commonly illustrate adherence problems in this age group. Antibiotic treatment often leads to rapid symptom relief, which may prompt adolescents to discontinue therapy prematurely once they feel better (Molan et al. 2025). This behavior is usually unintentional rather than deliberate opposition and reflects a disconnect between symptomatic improvement and the need for treatment completion. Adverse effects, inconvenient dosing schedules, and limited awareness of long-term consequences such as treatment failure or antimicrobial resistance may further contribute to early discontinuation.

Chronic conditions, in contrast, require sustained, often lifelong, engagement with therapy, rendering adherence challenges more complex and multifactorial. Asthma illustrates how fluctuating symptom burden may undermine adherence: during asymptomatic periods, adolescents may question the necessity of regular controller medication, particularly inhaled corticosteroids, while continuing to rely on short-acting bronchodilators for immediate relief (Desai and Oppenheimer 2011). This pattern reflects an interaction between disease intermittency, perceived medication efficacy, and the desire to minimize visible or stigmatizing treatment behaviors in social settings (Taddeo et al. 2008). Device-related factors, technique requirements, and concerns about side effects such as weight gain or growth suppression may further influence adherence decisions, even when objective disease control deteriorates.

Type 1 diabetes represents another chronic condition in which adherence is strongly shaped by disease visibility, regimen complexity, and psychosocial burden (Taddeo et al. 2008). Daily insulin administration, regular glucose monitoring, and dietary planning impose a continuous cognitive and behavioral load that may conflict with adolescents’ pursuit of normalcy and autonomy. Consistent with this burden, a study among adolescents with type 1 diabetes reported that 25% of patients were indifferent to insulin injections, 29% did not consistently monitor blood glucose levels, and as many as 81% did not adhere to dietary recommendations, highlighting the multidimensional nature of non-adherence in this population (Azar et al. 2024). Clinically, non-adherence may manifest as skipped insulin doses, irregular glucose monitoring, or deliberate dose adjustment, often driven by fear of hypoglycemia, weight-related concerns, or social discomfort. Importantly, these behaviors typically reflect intentional non-adherence rooted in competing developmental and social priorities rather than insufficient knowledge. As observed in both asthma and diabetes, effective adherence support therefore requires addressing not only treatment characteristics but also adolescents’ illness beliefs, perceived control, and the social meaning attributed to chronic disease management (Taddeo et al. 2008; Campagna et al. 2020; Azar et al. 2024).

## Multi-level recommendations to improve adherence in adolescents

Improving the effectiveness of pharmacotherapy in adolescence requires strategies that explicitly address adherence-related challenges within a broader developmental context. Medication use in this period cannot be guided solely by pharmacological principles but must also consider individual autonomy, psychosocial dynamics, and everyday treatment contexts (Kyngas et al. 2000; Taddeo et al. 2008; Sansom-Daly et al. 2012; Campagna et al. 2020; Farre et al. 2025). A multi-level framework for supporting rational medication use and adherence is outlined in Table 1.

**Table 1:** Key strategies to enhance medication adherence during adolescence

Level	Basic challenge in adolescence	Adherence-supporting strategies
Individual-focused	Limited insight, autonomy seeking	Involve adolescents in decision-making; explain treatment goals in age-appropriate language; verify understanding using teach-back
Clinician-patient	Trust and communication gaps	Establish an empathetic, trust-based relationship independent of caregivers; use shared decision-making
Therapy-related	Regimen complexity	Simplify dosing schedules; align administration with daily routines; if possible, prefer once-daily or long-acting formulations
Family and psychosocial	Tension between support and autonomy	Encourage supportive but non-coercive parental involvement; negotiate empathetically about sharing responsibilities
Health literacy	Safe self-management	Provide clear guidance on OTC use, adverse effects, and drug interactions; indicate when professional advice is needed
Digital context	Forgetfulness, motivation	Use reminder systems, calibrated and safe mobile apps, and brief digital educational tools embedded in care pathways

In conclusion, medication use during adolescence is more complex and multidimensional than in earlier childhood. Treatment adherence in this population is directly influenced by individual autonomy, psychosocial factors, peer dynamics, pharmaceutical formulation preferences, and patterns of medication access. Evidence indicates that adherence problems are particularly pronounced among adolescents with chronic diseases, adversely affecting treatment effectiveness and health outcomes. Addressing these challenges requires a comprehensive approach that integrates pharmacological considerations with developmental, psychological, and social dimensions of care. Framing adherence within a rational pharmacotherapy perspective allows adolescent-specific needs to be identified more accurately and supports meaningful engagement of young people in treatment processes. Rather than isolated interventions, layered and context-sensitive strategies across individual, therapeutic, and system levels are needed to promote sustained adherence and safe medication use during this critical developmental period.

## Data Availability

All source data for this work (or generated in this study) are available upon reasonable request.
